# Vitamin B1 and sepsis: a prospective single-center study

**DOI:** 10.3389/fnut.2026.1730024

**Published:** 2026-01-21

**Authors:** Wenbo Yan, Yushu Ma, Jingping Yang, Hongyan Wang, Tiewei Li

**Affiliations:** 1 Department of Clinical Laboratory, Zhengzhou Key Laboratory of Children’s Infection and Immunity, Henan Children’s Hospital, Children’s Hospital Affiliated to Zhengzhou University, Zhengzhou, Henan, China; 2 Respiratory and Critical Care Medicine Department, Inner Mongolia Baogang Hospital, Baotou, Inner Mongolia, China

**Keywords:** prevalence, sepsis, sequential organ failure assessment score, VB1, VB1 deficiency

## Abstract

**Purpose:**

Vitamin B1 (VB1), an essential coenzyme in cellular energy metabolism, is postulated to modulate clinical outcomes in sepsis. However, the relationship between VB1 and sepsis remains inadequately explored both domestically and internationally, and its potential clinical value remains unclear. Therefore, this study aims to investigate the association between VB1 deficiency and the severity of sepsis, and to evaluate the potential clinical utility of VB1 in ameliorating sepsis-induced organ injury and coagulation dysfunction.

**Methods:**

A total of 67 patients from Inner Mongolia Baogang Hospital were enrolled in this study. Among them, 41 were assigned to the sepsis group (clinically diagnosed with sepsis), and the remaining 26 comprised the control group (patients with ordinary pneumonia who did not meet the clinical diagnostic criteria for sepsis). Serum VB1 levels were measured using ultra-high-performance liquid chromatography–tandem mass spectrometry from peripheral blood samples. Clinical and laboratory data were obtained from electronic medical records. Multivariate logistic regression analysis was employed to assess the potential of VB1 as an independent biomarker for sepsis. All statistical analyses were performed using SPSS version 27.0.

**Results:**

The sepsis group exhibited significantly lower VB1 levels than the control group (*p* < 0.001). Further analysis revealed a significantly higher proportion of sepsis patients in the VB1 deficiency group compared to the control group, whereas the VB1 sufficiency group showed a significantly lower proportion of sepsis patients (*p* < 0.001). Correlation analysis demonstrated significant negative correlations between VB1 levels and sequential organ failure assessment scores, procalcitonin, D-dimer, creatinine, and cardiac troponin I, and positively correlated with albumin. Multivariable logistic regression analysis revealed that when VB1 was analyzed as a continuous variable, a high VB1 level was independently associated with a low prevalence of sepsis (OR = 0.127, 95% CI: 0.022–0.744, *p* = 0.022).

**Conclusion:**

VB1 levels exhibit an inverse association with key inflammatory biomarkers in patients with sepsis, and reduced VB1 concentration is independently associated with a higher prevalence of sepsis.

## Introduction

Sepsis is a life-threatening condition arising from a dysregulated host response to infection, leading to systemic inflammation and organ dysfunction, and posing a significant global public health challenge (1). Recent data from 2025 indicate that the overall mortality rate among sepsis patients is 35%, which increases to 66% in high-risk populations (2–4). Survivors of severe sepsis or septic shock frequently suffer from long-term disability and poor outcomes, largely attributable to tissue and organ injury caused by thrombosis, hypoperfusion, and inflammatory imbalance (5). In addition, sepsis is associated with profound metabolic dysregulation, including mitochondrial dysfunction, lactate accumulation-induced acidosis, and impaired adenosine triphosphate (ATP) synthesis (6). These disturbances compromise cellular energy metabolism and organ perfusion, thereby directly contributing to multi-organ failure (7, 8). This vicious cycle highlights the crucial role of metabolic dysfunction in driving sepsis-related inflammatory injury.

Vitamin B1 (VB1) is an essential coenzyme required for cellular energy metabolism. It serves as a key cofactor for the pyruvate dehydrogenase (PDH) and *α***-**ketoglutarate dehydrogenase (KGDH) complexes, central enzymes in aerobic glucose metabolism and the tricarboxylic acid (TCA) cycle (9). Previous research suggests that the beneficial effects of VB1 in sepsis are mediated through the correction of metabolic abnormalities, improved energy generation (10), reduction of lactate levels (10), and attenuation of oxidative stress (11, 12). Moreover, growing evidence indicates that VB1 may also play a role in modulating the inflammatory response in sepsis. For instance, it has been shown to lower C-reactive protein (CRP) levels in patients, suggesting an anti-inflammatory effect (13).

While existing evidence indicates that VB1 helps overcome sepsis-induced metabolic disturbances by providing essential metabolic support, thereby facilitating the restoration of homeostasis (14), its role in the inflammatory response remains underexplored. This study therefore aims to evaluate the association between VB1 levels and inflammatory indicators in sepsis, as well as its potential value for clinical.

## Materials and methods

### Study design and population

This prospective, single-center study was conducted at Inner Mongolia Baogang Hospital (Inner Mongolia, China). We enrolled 41 consecutive patients diagnosed with sepsis between August 2024 and April 2025. For comparison, a control group of 26 subjects with ordinary pneumonia (not meeting sepsis criteria) was recruited during the same period. The inclusion criteria for the sepsis group were: (1) age ≥ 18 years; (2) sepsis diagnosis within 24 h of intensive care unit (ICU) admission; (3) complete clinical and laboratory data at admission; (4) no use of VB1-containing medications during hospitalization. The control group (ordinary pneumonia) inclusion criteria were: (1) age ≥ 18 years; (2) clinical diagnosis of pneumonia; and (3) complete baseline data. Exclusion criteria for all subjects were: (1) declined to provide informed consent; (2) history of hematological diseases, malignancy, or autoimmune disorders; (3) incomplete clinical or laboratory data. The study protocol was approved by the Medical Ethics Committee of Inner Mongolia Baogang Hospital (Approval No: 2022-MER-110) and adhered to the Declaration of Helsinki. Written informed consent was obtained from all participants prior to enrollment.

### Clinical definition

According to the Third International Consensus Definitions for Sepsis and Septic Shock (Sepsis-3) (15), the diagnosis of sepsis was defined as follows: (1) evidence of microbial infection based on laboratory or imaging studies; (2) acute organ dysfunction, quantified by an increase in the sequential organ failure assessment (SOFA) score by ≥2 points from baseline. The SOFA score evaluates dysfunction in the respiratory (PaO₂/FiO₂), coagulation (platelet count), hepatic (bilirubin), cardiovascular (hypotension/vasopressor requirement), neurological (Glasgow Coma Scale), and renal (creatinine/urine output) systems. (3) Leukocytosis (>12 × 10^9^/L) or leukopenia (<4 × 10^9^/L). (4) Tachypnea with an oxygenation index (PaO₂/FiO₂) < 300 mmHg, and heart rate >90 beats per minute. The diagnostic criteria for common pneumonia were included and excluded according to the new guidelines for severe community-acquired pneumonia (16). Both pneumonia and sepsis were diagnosed by two investigators according to published international diagnostic criteria.

### Data collection

Patient clinical data collected from electronic medical records on the day of admission included age, sex, body mass index (BMI), body temperature, respiratory rate, SOFA score, pre-hospital medications, as well as laboratory parameters such as neutrophil count, procalcitonin (PCT), creatinine, D-dimer, fibrinogen (FIB), albumin (ALB), aspartate aminotransferase (AST), and cardiac troponin I (cTnI). In this study, PCT levels exceeding the measurable upper limit (>100 ng/mL) were defined as 101 ng/mL. Neutrophil count was measured using a MACCURA fully automated hematology analyzer (MACCURA Biotechnology, Sichuan, China). AST and creatinine levels were quantified using a Siemens 2,400 fully automated biochemical analyzer (Siemens Healthineers, Erlangen, Germany).

### Measurement of VB1

Serum VB1 levels were quantified by ultra-performance liquid chromatography–tandem mass spectrometry (UPLC-MS/MS) using a Waters Xevo TQ-S Micro system (Waters Corporation, Massachusetts, USA). To minimize VB1 degradation, the experimental protocol was strictly followed. Blood samples were centrifuged to separate serum within 30 min after collection. The isolated serum samples were immediately frozen and stored at −80 °C until analysis, with strict protection from light throughout the process. No freeze–thaw cycles were performed prior to UPLC-MS/MS analysis. Sample preparation was performed following the manufacturer’s protocol outlined in the commercial reagent kit (Shanghai Kehua Biological Technology Co., Ltd.). Analysis was carried out in the positive ion mode with electrospray ionization. The following instrument parameters were applied: capillary voltage, 3.0 kV; ion source temperature, 150 °C; desolvation temperature, 500 °C; desolvation gas flow, 1,000 L/h; and cone gas flow, 50 L/h. Internal quality control was maintained using calibrators and controls included in the reagent kit with each analytical run. The coefficient of variation for intra- and inter-assay precision was maintained below 10%. Data acquisition, peak identification, and quantification were performed using MassLynx software (Waters Corporation, Massachusetts, USA).

### Statistical analysis

Normally distributed data are presented as mean ± standard deviation (SD) and were compared using the *t*-test. Non-normally distributed continuous data are expressed as median (interquartile range) and were analyzed with the Mann–Whitney U test. Categorical variables are summarized as frequency (percentage) and were compared using the chi-square test. Spearman correlation analysis was employed to assess the relationships between VB1 and other clinical and laboratory parameters. Multivariable logistic regression analysis was performed to determine whether VB1 is an independently associated factor for sepsis. Variables with a *p*-value < 0.05 in the univariable logistic analysis were included in the multivariable logistic regression model. All statistical analyses were performed using IBM SPSS Statistics, Version 27.0 (SPSS Inc., Chicago, IL, USA). A two-sided *p*-value of less than 0.05 was considered statistically significant.

## Results

### Study population characteristics

**Figure 1:**
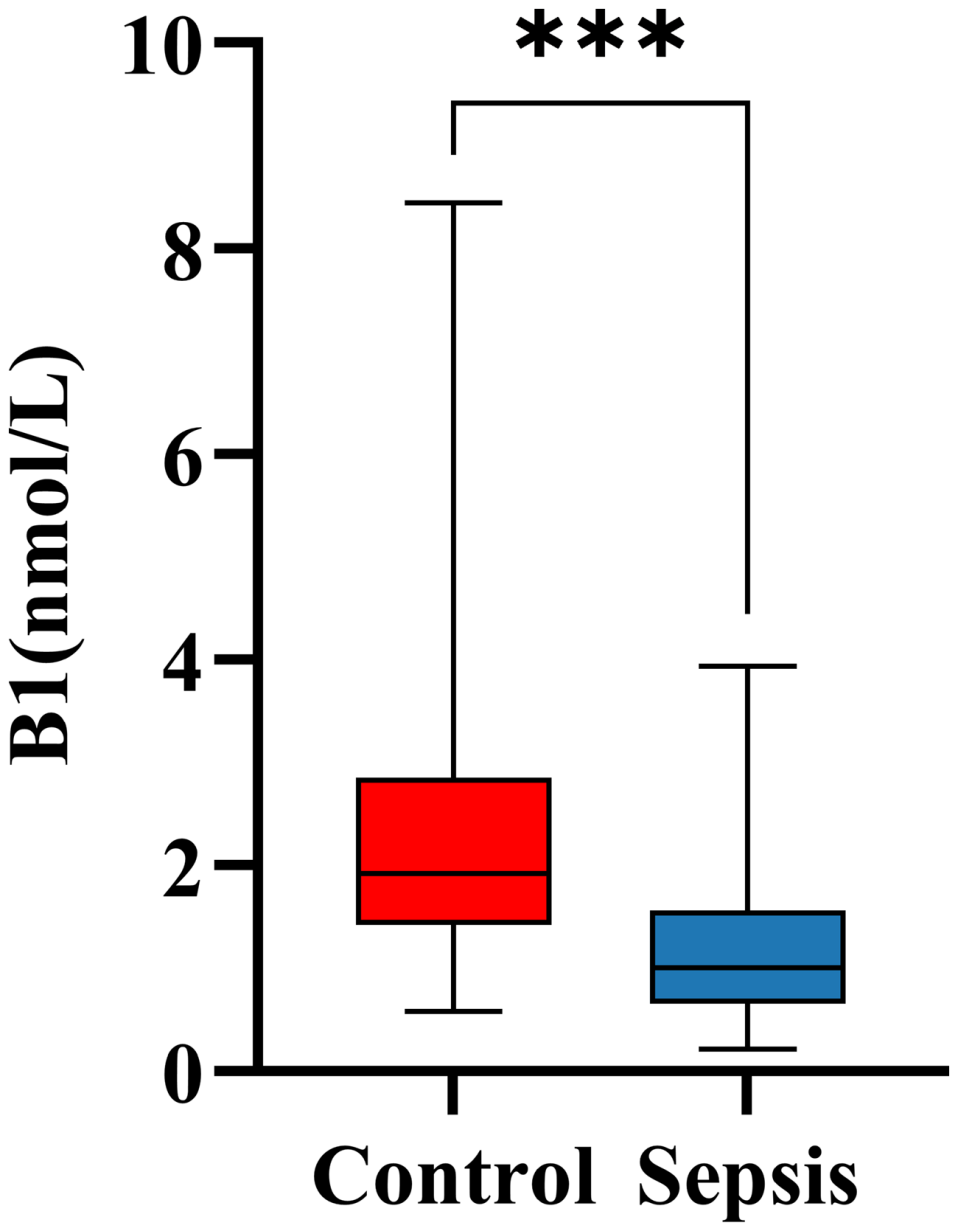
Differences in B1 levels between sepsis and control groups. ***p < 0.001; ns, no significant; B1, vitamin B1.

**Figure 2:**
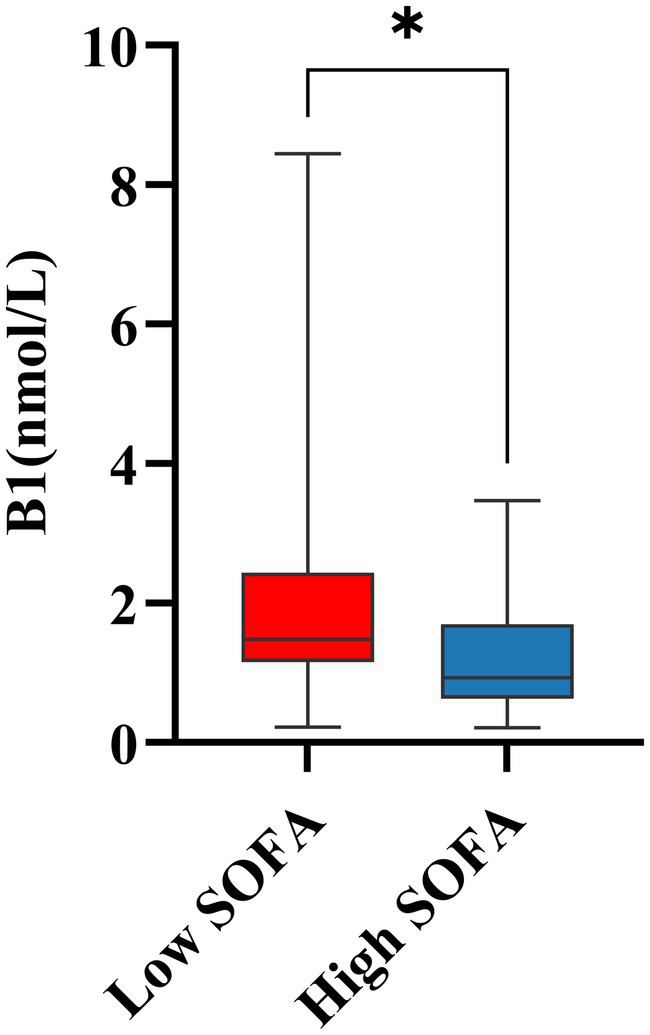
Differences in B1 levels between high and low SOFA scores. *p < 0.05; ns, no significant; B1, vitamin B1.

A total of 67 infected patients were enrolled in this study, comprising 39 males (58.2%) and 28 females (41.8%). Demographic characteristics and laboratory findings are presented in Table 1. Among the cohort, 41 patients were clinically diagnosed with sepsis, while the remaining 26 patients, diagnosed with non-septic pneumonia and not meeting the sepsis criteria, served as the control group. Compared with the control group, patients in the sepsis group exhibited significantly higher body temperature and respiratory rate; however, no significant differences were observed in age, sex, or BMI between the two groups. Biochemical analysis revealed that septic patients had significantly elevated levels of inflammatory markers, including neutrophil count, PCT, creatinine, D-dimer, AST, and troponin (*p* < 0.05). In contrast, ALB levels, which have been associated with VB1 deficiency, were significantly lower in the sepsis group (*p* = 0.005) (17), while fibrinogen levels showed no significant difference. Additionally, septic patients demonstrated significantly lower VB1 levels and higher SOFA scores (both *p* < 0.001). Based on the established reference range for VB1 (18), the 67 patients were stratified into two groups: a VB1 deficient group (< 1.56 nmol/L) and a VB1 sufficient group (≥ 1.56 nmol/L). Further analysis between the control and sepsis groups revealed a statistically significant difference in the distribution of VB1 status (*p* < 0.001), which is further illustrated in Figure 1. Furthermore, patients were categorized according to the median SOFA score (median = 3) into a high SOFA group (SOFA > 3) and a low SOFA group (SOFA ≤ 3). Further analysis of the high and low SOFA groups revealed that within the sepsis group, patients with high SOFA scores accounted for 65.85% of the total group, while those with low SOFA scores constituted only 34.15%. In the control group, high SOFA scores were observed in 3.85% of subjects, and low SOFA scores in 96.15%. A subsequent comparison of VB1 levels between the high SOFA and low SOFA groups was performed, with the results presented in Figure 2. Further analysis of preadmission medication use among the 67 included patients revealed no significant differences in the use of antidiabetic drugs, lipid-lowering agents, antihypertensive drugs, diuretics and hormonal drugs.

**Table 1:** Baseline characteristics of sepsis patients and controls.

Variables	Control (*n* = 26)	Sepsis (*n* = 41)	p*
Age (years)	74 (69, 83)	79 (73, 85)	0.360
Male, *n* (%)	15 (57.69%)	24 (58.54%)	0.947
BMI (kg/m^2^)	22.40 (19.58, 25.15)	21.25 (17.00, 25.39)	0.382
Temperature (°C)	36.50 (36.50, 37.10)	37 (36.55, 38.30)	**0.011**
Respiratory (rate/min)	21 (20, 21)	22 (21, 25)	**<0.001**
SOFA score	1 (0, 2)	5 (3, 9)	**<0.001**
SOFA status			**<0.001**
High SOFA	1 (3.85%)	27 (65.85%)	
Low SOFA	25 (96.15%)	14 (34.15%)	
Hematologic parameters			
Neutrophils (×10^9^/L)	5.42 (3.69, 9.21)	8.50 (5.24, 13.79)	**0.032**
PCT (ng/mL)	0.20 (0.06, 1.18)	2.07 (0.58, 6.36)	**<0.001**
Creatinine (μmol/L)	65.50 (52.35, 85.00)	94.70 (58.25, 154.25)	**0.036**
D-dimer (μg/mL)	0.68 (0.50, 0.83)	1.87 (1.06, 9.38)	**<0.001**
FIB (U/L)	4.66 ± 1.49	4.66 ± 2.09	0.992
Biochemical parameters			
ALB (g/L)	36.35 (30.83, 38.68)	30.80 (26.45, 35.10)	**0.005**
AST (U/L)	18.50 (14.75, 29.00)	30.00 (18.00, 59.00)	**0.006**
cTnI (ng/mL)	0.01 (0.01, 0.01)	0.03 (0.01, 0.13)	**<0.001**
VB1 (nmol/L)	1.93 (1.43, 2.86)	1.00 (0.66, 1.56)	**<0.001**
VB1 status			**<0.001**
VB1 sufficiency, *n* (%)	18 (69.2%)	10 (24.4%)	
VB1 deficiency, *n* (%)	8 (30.8%)	31 (75.6%)	
Medications, *n* (%)			
Antidiabetic drugs	5 (19.23%)	3 (7.32%)	0.146
Lipid-lowering agents	5 (19.23%)	6 (14.63%)	0.623
Antihypertensive drugs	12 (46.15%)	11 (26.83%)	0.107
Diuretics	1 (3.85%)	3 (7.32%)	0.562
Hormonal drugs	3 (11.54%)	2 (4.88%)	0.316

^*^Boldface highlights results with statistically significant differences.

BMI, Body Mass Index; SOFA, Sequential Organ Failure Assessment; PCT, Procalcitonin; FIB, Fibrinogen; ALB, albumin; AST, Aspartate aminotransferase; cTnI, cardiac troponin I; VB1, Vitamin B1.

### Comparative analysis of clinical parameters by VB1 status

Further statistical analysis was performed to evaluate the association between different VB1 levels and various clinical and demographic parameters. As shown in Table 2, compared with the VB1 sufficiency group, the VB1 deficiency group exhibited significantly higher PCT levels and SOFA scores (*p* < 0.05). The prevalence of sepsis decreased significantly from 79.49% in the VB1 deficiency group to 35.71% in the VB1 sufficiency group (*p* < 0.001). Conversely, the control group was predominantly distributed in the VB1 sufficiency group (64.29%), with only 20.51% in the VB1 deficiency group (*p* < 0.001). ALB was also significantly higher in the VB1 sufficient group than in the VB1 deficient group (*p* = 0.011). However, no statistically significant differences were observed between the two VB1 groups in terms of age, male, BMI, neutrophil count, D-dimer, FIB, cTnI, or AST.

**Table 2:** Clinical and demographic characteristics based on the VB1.

Variables	VB1 sufficiency (≥1.56 nmol/L) (*n* = 28)	VB1 deficiency (<1.56 nmol/L) (*n* = 39)	p*
Age (years)	78 (70, 87)	79 (72, 83)	0.990
Male, *n* (%)	14 (50.0%)	25 (64.1%)	0.255
BMI (kg/m^2^)	21.55 (17.88, 24.40)	22.30 (18.70, 26.60)	0.708
SOFA score	2 (0, 4)	3 (2, 8)	**0.005**
PCT (ng/mL)	0.28 (0.09, 3.67)	1.39 (0.40, 6.55)	**0.007**
Neutrophils (×10^9^/L)	5.83 (4.64, 10.91)	6.96 (4.25, 13.79)	0.431
D-dimer (μg/mL)	0.84 (0.54, 1.74)	1.31 (0.67, 3.93)	0.106
FIB (U/L)	4.68 (3.28, 5.82)	4.93 (3.77, 5.82)	0.855
ALB (g/L)	35.05 (30.83, 38.50)	30.80 (26.80, 36.60)	**0.011**
AST (U/L)	21 (16, 32)	26 (16, 44)	0.434
cTnI (ng/mL)	0.011 (0.011, 0.017)	0.013 (0.011, 0.089)	0.096
Clinical data			**<0.001**
Control, *n* (%)	18 (64.29%)	8 (20.51%)	
Sepsis, *n* (%)	10 (35.71%)	31 (79.49%)	

*Boldface highlights results with statistically significant differences.

VB1, Vitamin B1; BMI, Body Mass Index; SOFA, Sequential Organ Failure Assessment; PCT, Procalcitonin; FIB, Fibrinogen; cTnI, cardiac troponin I; ALB, albumin; AST, Aspartate aminotransferase.

### Correlation between VB1 and clinical parameters

To further explore the relationship between VB1 and clinical parameters, a correlation analysis was conducted. As shown in Table 3, VB1 levels exhibited a significant negative correlation with PCT (*r* = −0.475, *p* < 0.001), a marker of infectious inflammation. VB1 level was positively correlated with ALB (*r* = 0.393, *p* = 0.001), a marker of nutritional status. Significant negative correlations were also observed between VB1 and the coagulation biomarker D-dimer (*r* = −0.338, *p* = 0.005), the renal function biomarker creatinine (*r* = −0.268, *p* = 0.028), the myocardial injury marker cTnI (*r* = −0.406, *p* = 0.001), and the SOFA score (*r* = −0.476, *p* < 0.001), which reflects the severity of infection and inflammation. In contrast, no significant correlations were found between VB1 levels and age, sex, BMI, temperature, respiratory rate, neutrophil count, FIB, or AST.

**Table 3:** Correlations between the VB1 and clinical parameters.

Variables	*r*	p*
Age (day)	−0.040	0.746
Male, *n* (%)	0.184	0.136
BMI (kg/m^2^)	−0.010	0.934
Temperature (°C)	−0.071	0.568
Respiratory (rate/min)	−0.010	0.933
Neutrophils (×10^9^/L)	−0.212	0.084
PCT (ng/mL)	−0.475	**<0.001**
D-dimer (μg/mL)	−0.338	**0.005**
FIB (U/L)	−0.048	0.704
AST (U/L)	−0.132	0.288
ALB (g/L)	0.393	**0.001**
Creatinine (μmol/L)	−0.268	**0.028**
cTnI (ng/mL)	−0.406	**0.001**
SOFA score	−0.476	**<0.001**

*Boldface highlights results with statistically significant differences.

BMI, Body Mass Index; PCT, procalcitonin; FIB, Fibrinogen; ALB, albumin; AST, Aspartate aminotransferase; cTnI, cardiac troponin I; SOFA, Sequential Organ Failure Assessment.

### Predictive value of VB1 for the presence of sepsis

Multivariable binary logistic regression was performed to assess whether VB1 is an independently associated factor for sepsis. Variables with a *p*-value < 0.05 in the univariate regression analysis—including body temperature, respiratory rate, D-dimer, AST, and neutrophil count—were incorporated into the multivariate model. As presented in Table 4, when analyzed as a continuous variable, VB1 was independently associated with sepsis (OR = 0.127, 95% CI: 0.022–0.744, *p* = 0.022). In categorical analysis, patients with VB1 deficiency were associated with 50.0-times higher odds of sepsis compared to those with sufficient VB1 levels (OR = 50.017, 95% CI: 2.905–861.189, *p* = 0.007).

**Table 4:** Regression analyses to determine the independent predictor of sepsis.

Variables	Univariate		Multivariate #	
	OR (95% CI)	p*	OR (95% CI)	p*
VB1 (nmol/L)	0.371 (0.201–0.686)	**0.002**	0.127 (0.022–0.744)	**0.022**
VB1 status				
VB1 sufficiency (≥1.56 nmol/L)	1		1	
VB1 deficiency (<1.56 nmol/L)	6.975 (2.331–20.875)	**<0.001**	50.017 (2.905–861.189)	**0.007**

*Boldface highlights results with statistically significant differences. #Adjusted for temperature, respiratory rate, D-dimer, AST, neutrophils count.

AST, Aspartate aminotransferase; VB1, Vitamin B1.

## Discussion

Sepsis, as a systemic inflammatory response, is often accompanied by metabolic disturbances and increased energy expenditure (19). The pathophysiological process of sepsis is highly complex, primarily involving enhanced glycolysis, mitochondrial dysfunction, excessive ROS production (20), and the resulting oxidative stress and tissue damage (21, 22). Early metabolic alterations in sepsis include the accumulation of pyruvate and its conversion to lactate, leading to increased tissue levels of lactate and glycerol, as well as enhanced anaerobic oxidation (21). Concurrently, sepsis involves mitochondrial dysfunction and increased ROS levels, which readily induce heightened oxidative stress and insufficient ATP supply, further contributing to patient shock and multiple organ failure (21, 22). Sepsis is a disease caused by severe infectious diseases, with pneumonia being one of the most common infectious etiologies of sepsis (23). A study by Khalid et al. found that VB1, as an adjuvant therapy, significantly reduced the in-hospital mortality rate in pneumonia patients (24). Therefore, VB1, as a coenzyme in the body’s metabolism, still holds potential clinical value in anti-inflammatory effects and improving sepsis.

In the study by Xu et al. (10), it was confirmed that VB1 primarily alleviates the inflammatory response in sepsis treatment by modulating metabolic processes and subsequently regulating inflammatory cytokine levels. This approach has been applied in clinical practice. However, systematic reviews of its efficacy reveal heterogeneity in the existing evidence: some studies reported improvements in patient outcome scores (e.g., SOFA score) or critical illness indicators (e.g., CRP), while others failed to demonstrate significant benefits regarding ICU length of stay, need for mechanical ventilation, or mortality (25). Furthermore, the study by Khan et al. (26) found that VB1 can be used as an adjunct in managing sepsis-related complications (such as Wernicke’s encephalopathy). When combined with other vitamins and trace elements, it may improve metabolic disturbances, nutritional deficiencies, and neurological complications induced by sepsis, thereby reducing sepsis-induced parenchymal organ damage. In our study, we also found that VB1 levels were significantly negatively correlated with patient creatinine and cTnI levels. Therefore, sepsis patients may develop VB1 deficiency due to inadequate intake, increased metabolic demands, or accelerated renal clearance, potentially exacerbating lactic acidosis and organ damage (e.g., myocardial and renal injury) (13, 27). Although the present study confirmed an association between low VB1 levels and sepsis severity, it did not incorporate indicators such as lactate into the analysis. Furthermore, while multivariable logistic regression showed a significant independent association between VB1 deficiency and sepsis compared to patients with sufficient VB1 levels, the confidence interval for the VB1 deficientcy was extremely wide (spanning an order of magnitude), indicating unstable estimates likely resulting from low event counts in this group. Thus, the pathophysiological mechanisms linking VB1 levels to sepsis and its related complications—including lactic acidosis and organ injury—as well as the therapeutic efficacy of VB1 monotherapy in improving outcomes in sepsis and its complications, require further validation through high-quality studies. Nonetheless, these findings further highlight the potential clinical relevance of VB1 in sepsis and its associated complications.

Currently, the relationship between VB1 and sepsis remains an area of limited and inconsistent evidence. The study by Moskowitz and Donnino (27) highlighted that VB1 deficiency is relatively common in sepsis patients, particularly among high-risk groups such as those with malnutrition, and suggested that supplementation may improve lactic acidosis and organ injury. However, that study also acknowledged that the effect of VB1 may be limited by significant inter-individual variability. In contrast, Mishra et al. (13) directly compared high-dose VB1 with vitamin C (VC) in sepsis patients. While VC is a recognized antioxidant with known anti-inflammatory and organ-protective properties (28), VB1 performed comparably to VC in reducing CRP levels, indicating a potential role in modulating the sepsis-induced inflammatory response. Nevertheless, in subsequent analyses, VB1 was inferior to VC in improving SOFA scores or reducing vasopressor duration, with no significant improvement observed in the VB1 group.

These observations lead to two pertinent considerations regarding the inconsistent findings across studies. First, the present study identified a significant correlation between VB1 levels and SOFA score but not with other clinical indicators, whereas other reports have shown divergent associations (25, 28). This discrepancy may be partly explained by the nature of the SOFA score as a composite measure of overall disease severity—higher scores reflect more critical illness. In line with this, our data demonstrated a significant negative correlation between VB1 levels and SOFA score. Moreover, the sepsis group exhibited significantly higher SOFA scores than the control group (patients with ordinary pneumonia), and lower VB1 levels were consistently observed in patients with higher SOFA scores, a pattern further confirmed in the VB1-deficient subgroup. Thus, lower VB1 levels in patients with higher SOFA scores may reflect or contribute to worsened outcomes through impaired cellular energy metabolism (29, 30). Second, VB1 primarily functions as a coenzyme in energy metabolism rather than as a direct anti-inflammatory or organ-protective agent. Its measurable effects in sepsis may therefore be modulated by various patient-specific factors, including age, sex, lifestyle, and BMI (31), which could account for the variability across different study populations and designs.

It must be emphasized that, as a prospective single-center study, our research cannot establish a causal relationship between VB1 levels and outcomes in sepsis. The observed association may be interpreted in two ways: pre-existing VB1 deficiency could impair mitochondrial function and increase susceptibility to sepsis and poorer outcomes (32); alternatively, the hypermetabolic state and inflammatory response during sepsis may rapidly deplete VB1 stores, making low VB1 a marker of severity rather than a causative factor (14). To clarify this relationship, further investigations are necessary—such as dynamic monitoring of VB1 and its metabolites in early sepsis or high-risk cohorts, or interventional randomized controlled trials assessing whether VB1 supplementation can improve clinical outcomes.

Sepsis patients commonly present with coagulation dysfunction and disseminated intravascular coagulation (DIC) (33, 34). Previous studies generally considered VB1 to be related to metabolism, with no direct link to coagulation parameters. Interestingly, our study found that D-dimer levels were significantly different between the sepsis and control groups, and VB1 levels were negatively correlated with D-dimer levels. In contrast, FIB showed no statistically significant correlation. VB1 deficiency can disrupt energy metabolism, including glucose metabolism, thereby impairing hepatic function (35, 36). As the primary site of coagulation factor synthesis, liver dysfunction may disturb coagulation homeostasis and lead to coagulation abnormalities (36). Moreover, metabolic disorders are often accompanied by inflammation or oxidative stress, which can activate coagulation pathways and alter hemodynamics, ultimately elevating D-dimer levels (27). As mentioned in the study by Fujii et al. (37), vitamin supplementation in sepsis might improve vascular function by reducing oxidative stress, but this does not directly equate to changes in coagulation parameters. Whether VB1 directly affects coagulation parameters requires further investigation.

In this study, we conducted a first-of-its-kind comparison between patients with sepsis and non-septic patients with ordinary pneumonia infection to assess the association between VB1 levels and sepsis. Our data demonstrated a significant intergroup difference in VB1 levels between the control and sepsis groups. Further analysis revealed that sepsis patients had lower VB1 levels compared to the control group. Additional analyses showed negative correlations between VB1 levels and D-dimer, PCT, SOFA score, creatinine, and troponin I—indicators related to coagulation, inflammation, and organ injury. VB1 level was positively correlated with ALB. Moreover, multivariable logistic regression analysis revealed that VB1 levels were independently associated with sepsis in the adult study population.

This study has several limitations. First, this single-center study had a limited sample size and primarily focused on an adult patient with sepsis. Future research with larger-scale, more age-diverse samples is necessary to obtain a more precise assessment of the relationship between VB1 and sepsis. Second, while selecting patients with ordinary pneumonia as the control group aimed to compare against individuals with less severe infection than sepsis—thereby minimizing the influence of general acute-phase response following infection, but this choice itself may introduce confounding factors. The inflammatory milieu of pneumonia may also affect VB1 levels, potentially attenuating the observed between-group differences. Finally, as a single-center study, despite accounting for multiple variables such as anthropometric measures (e.g., BMI) and biochemical markers (e.g., albumin), we lacked other essential data including detailed dietary intake history, lifestyle, socioeconomic status, as well as follow-up information on subsequent treatments and long-term outcomes. It must be emphasized that as VB1 is a coenzyme for pyruvate dehydrogenase, lactate serves as a direct clinical biomarker of thiamine-related metabolic dysfunction. Unfortunately, due to incomplete lactate data in our prospective dataset, we were unable to include lactate in the correlation or regression analyses, which limits the strength of the metabolic argument. Future studies should prioritize the inclusion of lactate measurements. Consequently, we were unable to clearly distinguish between nutritional deficiency and the acute metabolic effects of critical illness. This limitation also hindered our ability to evaluate long-term prognosis or establish causality. These points underscore the need for more comprehensive nutritional data collection in future studies.

## Conclusion

In summary, this prospective single-center study demonstrates that circulating VB1 levels at admission are significantly lower in patients with sepsis compared to those with ordinary pneumonia. Furthermore, correlation analysis revealed that low VB1 levels are significantly associated with inflammatory markers and disease severity (SOFA score). Concurrently, low VB1 levels showed a significant correlation with a higher prevalence of sepsis. These findings suggest that maintaining sufficient VB1 levels may have potential clinical implications for preventing severe infection in sepsis, though whether supplementation to achieve this can prevent severe infection or improve outcomes in sepsis patients awaits confirmation from future intervention trials.

## Data Availability

The original contributions presented in the study are included in the article/supplementary material, further inquiries can be directed to the corresponding authors.
